# ‘There's only so much you can be pushed’: Magnification of the maternity staffing crisis by the 2020/21 COVID‐19 pandemic

**DOI:** 10.1111/1471-0528.17203

**Published:** 2022-05-26

**Authors:** Sarah Cordey, Gill Moncrieff, Joanne Cull, Arni Sarian, the ASPIRE‐COVID 19 Collaborative Group

**Affiliations:** ^1^ School of Community Health and Midwifery, THRIVE Centre University of Central Lancashire Preston UK; ^2^ Research in Childbirth and Health Group, THRIVE Centre University of Central Lancashire Preston UK; ^3^ School of Medicine University of Central Lancashire Preston UK

## Abstract

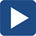
 This article includes Author Insights, a video abstract available at: https://vimeo.com/bjogabstracts/authorinsights17203

1

Concerns about the impact of staffing shortages and burnout in the maternity workforce on safe and respectful care are long‐standing, in the UK and internationally.^1^, ^2^ The COVID‐19 pandemic has further reduced workforce availability worldwide.^3^ We explored the impact of the pandemic on maternity staff experience.

We thematically analysed in‐depth interviews (November 2020–October 2021) with 28 frontline maternity staff and 28 heads of service from seven geographically and demographically diverse NHS Trusts in England, as part of the ASPIRE COVID‐19 study.

The pandemic magnified existing problems within maternity care. Well established challenges such as short staffing, organisational demands, and barriers to providing relational care were exacerbated by the pandemic, leaving staff emotionally exhausted and unable to carry on. While the service is usually maintained through the goodwill of its workers, this is not sustainable in the long‐term or through crisis situations. We identified three sub‐themes (Figure 1) that capture changing experiences as the pandemic progressed.

**FIGURE 1:**
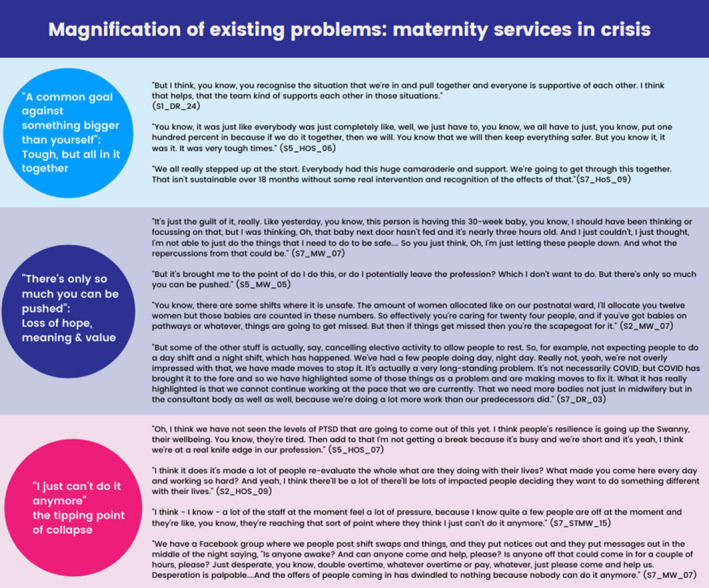
Themes developed through analysing the interview data with example quotes (for the full table of themes and quotes see File S1)

A sudden influx of staff and resources early in the pandemic, combined with a sense of camaraderie, public support and professional pride, led to an unexpectedly positive work environment. Despite fears for their own health and worries about taking home the virus to their families, many reported that making huge personal sacrifices was part of being involved in something bigger than themselves.

However, these protective factors did not last. Experiences later in the pandemic included poor staff recruitment and retention, deteriorating physical and psychological wellbeing, insufficient staffing and unmanageable workloads. Some described unsafe working practices such as an inability to provide one‐to‐one care in labour, and excessively long working hours. Many described the emotional distress of working intensively to maintain standards of care but feeling only able to do the ‘bare minimum’. For some, the dissonance between the safe and personalised care they wanted to provide, and the experience of dangerously low levels of staffing, was associated with accounts of significant moral injury and distress.

Those interviewed later in 2021 reported increasingly critical staffing shortages. Respondents described compassion fatigue, both towards their colleagues and for those in their care. ‘Exhausted’, ‘broken’, ‘unable to carry on’ or similar terms were used by a majority of participants. Serious concerns were raised about a rising incidence of burnout and breakdown, leading to an exodus of experienced and expert staff. One obstetrician warned of the ‘the biggest midwifery crisis of all time’.

Our findings indicate that the COVID‐19 pandemic has magnified the existing and escalating maternity staffing crisis in England, impacting on the ability to provide both safe and personalised care. International evidence suggests that maternity services globally face similar challenges.^3^ Coping mechanisms that usually enabled staff to go ‘above and beyond’ to plug service gaps were breaking down towards the end of the data collection period, reducing the sustainability of all but basic care, and risking the psychological, emotional and physical health of respondents.

The impact of sub‐optimal staffing on service user safety is increasingly highlighted in maternity safety reviews, which have also recognised that although staff are frequently intensely concerned about staffing ratios, these concerns have been dismissed.^4^, ^5^ Addressing insufficient staffing in maternity is a central recommendation of these reviews and can no longer be ignored.

There is a unique opportunity for a post‐pandemic rebuild of maternity services. This should begin by examining protective factors and organisational and political drivers that sustain psychological and physical staff wellbeing, and optimal service user outcomes and experiences. These include explicit organisational commitment to safe and sustainable staffing, flexible, autonomous practice, and protected time to provide person‐centred, relational care. Getting these factors right, may promote sustainable recruitment and retention of professional maternity care staff, both for care under normal circumstances and for future crises.

## Supporting information


File S1

File S2

Appendix S1

Appendix S2

Appendix S3

Appendix S4
