# Supplementary material for: ‘There's only so much you can be pushed’: Magnification of the maternity staffing crisis by the 2020/21 COVID‐19 pandemic
Source: BJOG. 2022 May 26;129(8):1408–9. doi: 10.1111/1471-0528.17203 (PMC9321880; doi:10.1111/1471-0528.17203)
Supplement: Supplementary file 1 — File S1 [file BJO-129-1408-s001.docx]

**Full table: themes developed through analysing interview data with example quotes**

| Theme | Quotes |
| --- | --- |
| “A common goal against something bigger than yourself”: Tough, but all in it together | It's weird when you're on labour ward, and you're in this room, with a woman in labour and you just have the baby because the whole rest of the world kind of crazy, but babies are still being born. Like it just carries on. (S7_MW_ 07)  But I think, you know, you recognise the situation that we're in and pull together and everyone is supportive of each other. I think that helps, that the team kind of supports each other in those situations (S1_DR_24)  I think as a team with colleagues, it's probably about different understanding and a more willingness to help each other out. So on the shop floor, I think that's definitely changed. And we're looking out for each other I suppose. Not that we didn't in the first place, but more, I think everyone's bonded in a different way because of the added pressures (S2_MW_07)  You know, it was just like everybody was just completely like, well, we just have to, you know, we all have to just, you know, put one hundred percent in because if we do it together, then we will. You know that we will then keep everything safer. But you know it, it was it. It was very tough times (S5_HOS_06)  The other thing that went out the window was all the meetings. It was just let's focus on maternity care in the COVID pandemic and that what, again, that was so whilst the service obviously wasn't moving forward at that point, but it was we were concentrating on the job being done and actually the women got really good care, really good care (HoS_SU_04)  We all really stepped up at the start. Everybody had this huge camaraderie and support. We're going to get through this together. That isn't sustainable over 18 months without some real intervention and recognition of the effects of that.” (S7_HoS_09) |
| “There's only so much you can be pushed”: Loss of hope, meaning & value | I mean, it's the latest real big focus on being efficient. They must be, we must be, efficient. We are busy. There is not enough people. We must be efficient. You must be. So we must have the baby, and then we expect you, when you come out the room, you've got all your notes up to date because someone's going to do your computer stuff. So that this person can go home or can go upstairs to the ward within the next hour and you just think what? You get, Knock, knock, knock when you’re suturing or something. Are your notes up to date? No. Oh well, if you've got the timings, we're on the CTG perfect. I'll just come in and do it? And you just think, Oh, there's no kind of care. (S7_MW_07)  I think PPE has had a really big impact because I think it makes the women feel even more distanced from us. And I think we've always we always have quite close relationships with our women. I think it's hard to forge those at the moment because they can't see or interact with you like they used to you. Simple things like a hug has now gone, you know you get ladies through and sometimes you give them a bit of a hug and you can't do that now. (S1_MW_22)  But when you've got midwives, you know, run off their feet trying to provide like the basic care for women and meet the safety standards. It's very difficult to go the extra mile just because you haven't got time and you haven't got the hands. (S7_STMW_15)  And we've managed on the staff that we have. But I feel that the atmosphere within the hospital has been, become, negative. Because each unit is pulling, trying to pull as many midwives as they possibly can. So we're just struggling in terms of the negativeness, the culture within the hospital. Because we haven't got the staff to go around. (S1_MW_07)  One person rang me 4 times in the same shift with the same problem and 4 times I had to say I am really sorry but... And in the end, you can't help but start to become annoyed but at the same time she's that worried she has rung 4 times. Then I feel guilty that I'm annoyed with her cause the pressure at work is so bad. So yeah. It's been really. It's been hard. (S1_MW_22)  It's just the guilt of it, really. Like yesterday, you know, this person is having this 30-week baby, you know, I should have been thinking or focussing on that, but I was thinking, Oh, that baby next door hasn't fed and it's nearly three hours old. And I just couldn't, I just thought, I'm not able to just do the things that I need to do to be safe.... So you just think, Oh, I'm just letting these people down. And what the repercussions from that could be (S7_MW_07)  I just think if they had more time, we would have like the appointments wouldn't just be very like "Oh, here's the heartbeat, how you doing, that's fine, bye" That's what it felt like, if they just had a bit more time to sort of get to know me, sort of, yeah, sort of build that relationship instead of it just being sort of like a very clinical appointment and then the next mum comes in (S6_SU_14)  You're on your own, pandemic or not, you want people that are the professionals. And not make you feel like you're an inconvenience and they haven't got time for you, which is, I don't think it's any fault of their own. I think maybe they're understaffed. But it wasn't a positive experience afterwards. (S4_SU_18)  But it's brought me to the point of do I do this, or do I potentially leave the profession? Which I don't want to do. But there's only so much you can be pushed. (S5_MW_05)  But some of the other stuff is actually, say, cancelling elective activity to allow people to rest. So, for example, not expecting people to do a day shift and a night shift, which has happened. We've had a few people doing day, night day. Really not, yeah, we're not overly impressed with that, we have made moves to stop it. It's actually a very long-standing problem. It's not necessarily COVID, but COVID has brought it to the fore and so we have highlighted some of those things as a problem and are making moves to fix it. What it has really highlighted is that we cannot continue working at the pace that we currently. That we need more bodies not just in midwifery but in the consultant body as well as well, because we're doing a lot more work than our predecessors did. (S7_DR_03)  For the consultants, we don't have a fully staff either. The juniors don't have a fully staffed roster. The fact that some of our juniors got pulled, actually not our obstetric trainees, but some of our juniors got pulled to the COVID wards and the rest of it put extra stress on them. Some of the, you know, the staffing in the rest of the hospital didn't necessarily come up to the idea that they were going to need to permanently staff certain areas, and they haven't really thought about that and they still haven't really covered that. So they're still asking for support for the rest of the area. And some of that's down to the fact that we try to get people in. We can't, you know, there is the, you know, no matter how hard you try, sometimes you just don't get the people you need. (S7_DR_03)  I think, to be quite honest, the pandemic's come at a time when we were probably at a national shortage of midwives. We had stress before 18 months ago we were short of staff. So you know, I know, so even probably before the pandemic hit, I think we were short of staff. So it just cemented it even more, really, I think the unit was run on goodwill, and I think to a degree, it still is. (S6_HCP_05)  I think we probably you know, again, it's a safety aspect, but people pulled out all the stops and worked. And I think people are tired. You know, I think that they we run risk if we're not careful, if this goes on for too much longer, that people will get very disillusioned, burnt out and, you know, not want to keep doing it (S1_HoS_06)  You know, there are some shifts where it is unsafe. The amount of women allocated like on our postnatal ward, I'll allocate you twelve women but those babies are counted in these numbers. So effectively you’re caring for twenty four people, and if you've got babies on pathways or whatever, things are going to get missed. But then if things get missed then you're the scapegoat for it (S2_MW_07) |
| “I just can't do it anymore”: the tipping point of collapse | I think, to be quite honest, the pandemic's come at a time when we were probably at a national shortage of midwives. We had stress before 18 months ago we were short of staff. So you know, I know, so even probably before the pandemic hit, I think we were short of staff. So it just cemented it even more, really, I think the unit was run on goodwill, and I think to a degree, it still is. So, yeah, I think it put a lot of stress, I work with a really excellent team and you know, lots of people did come in and do extra shifts and yeah, they were brilliant team, really (S6_HCP_05).  Just in the last few months, I suppose the staffing because we haven't had that extra input that we did right at the beginning. I suppose sort of managers and support, not support, specialist midwives have now gone back to their roles and whereas before, I think it was all hands-on deck. What do you need? and staff actually coming up and saying, I will do an extra shift, or I will be on call this night if you need me to be. But it's just not sustainable at all. And as it's going on, that's all sort of dwindled away a little bit more. So, the poor staff that were there are still there, they're still doing it with, sort of, no added support anymore. I think it's just sort of become the norm. But it still is really, really hard work for them (S4_MW_02)  I think - I know - a lot of the staff at the moment feel a lot of pressure, because I know quite a few people are off at the moment and they're like, you know, they're reaching that sort of point where they think I just can't do it anymore (S7_STMW_15)  As soon as you start cutting midwives out, you start piling more pressure on an individual midwife, making that midwife leave and then you just have a massive snowball effect, you're left with people that have no choice but to carry on because they can't possibly think what would happen if they also left. And then what? You know what you left with and left with midwives doing the bare minimum because they can't think of doing anything else and people don't get the care that they should (S7_MW_07)  So it's like I say, it's been really tricky with having had Continuity thrown into the mix. It because the staff definitely are not, you know, haven't been in a good place. But I think that is the impact of both Continuity and Covid, and the combination of the two was just too much for a lot of people (S7_MW_16)  It's been it's not just it's not just been the pandemic. There's been several things that have happened within the trust all at the same time, which ideally we would have postponed. I think continuity needed to happen. I mean, a massive advocate of continuity of carer but there was that the pandemic, we changed our electronic records system, we have massive amount of sickness. We had massive amount of shielders and it's now, you know, it's I don't know I don't know how we're going to get back. It has really affected the morale and people are leaving and it's I think it was too many things all at once, and I think that the thing with the continuity, we did sort of slow things down to start with, and then we had to try to regenerate the whole thing again. And everyone's just now feeling I can't do it anymore. There's too many things happening and I think the midwives completely understands why it needs to needs to happen. They're not opposed to it happening. They're just opposed to it happening right now when they're all feeling the way that they're feeling (S2_HOS_09)  The midwives who have been here for 20 years have just been through this huge change and then we're telling them now everything else is changing as well. It's like it's too much so and people are looking outside of the organisation for other jobs....... whether it's the pandemic continuity other changes, whether it's just the whole package. That's what's happening. We're not going to be left with a lot of very experienced midwives. I think we knew that before the pandemic because of the age we all are (laughs...). But I think this is like the icing on the cake (S2_HOS_09)  People are so tired. They're so tired and they still haven't had a holiday, and it's relentless, it's not going away, it's still there, and people's resilience is and activities back to like everything's worked, you know, in place. Like all of our quality improvement projects, the expectations are all back, the deadlines are back, all the work is being expected to be done and everyone is very tired (S5_HOS_02)  Oh, I think we have not seen the levels of PTSD that are going to come out of this yet. I think people's resilience is going up the Swanny, their wellbeing. You know, they're tired. Then add to that I'm not getting a break because it's busy and we're short and it's yeah, I think we're at a real knife edge in our profession (S5_HOS_07)  I think unfortunately the NHS runs on the goodwill of people, and that's how it manages to keep going in the way that it keeps going. And I worry the future is quite bleak because I think this has harmed quite a lot of people in terms of their view of their role, their future in the NHS (S5_MW_04)  I think it does it's made a lot of people re-evaluate the whole what are they doing with their lives? What made you come here every day and working so hard? And yeah, I think there'll be a lot of there'll be lots of impacted people deciding they want to do something different with their lives (S2_HOS_09)  We're still short staffed, we've still got sickness We've still got people who are broken. And I think as a consultant, it's quite often useful to say we've had I've had one midwife crying in the toilets when I went in... And you know, you go in and you go, Are you OK? Clearly, you're not okay. Don't lie to me. You're not okay.... We've all been there. We have all cried in toilets. Yeah, I don't know, I don't know a single person in medicine, full stop, who has not cried in a toilet. (S7_DR_03)  You know, not just, you know, nobody's got anything left. People working well above their hours. There's no staff. I heard there was something like 40 percent of staff off sick. We've got loads retiring who are taking early retirement. They aren’t going to be there anymore and they just keep talking about the fact we've got 17 new band fives and eight new Band six are starting. As if that's a good thing. (S7_MW_07)  I think - I know - a lot of the staff at the moment feel a lot of pressure, because I know quite a few people are off at the moment and they're like, you know, they're reaching that sort of point where they think I just can't do it anymore. I don't know what could be done, but I think just supporting them, I think. Mental health support for staff would be key, because I think that's what a lot of people struggled with was, you know? It was with mental health rather than like COVID and isolation and things, I think that's where people could have done with a bit more support. (S7_STMW_15)  We have a Facebook group where we people post shift swaps and things, and they put notices out and they put messages out in the middle of the night saying, "Is anyone awake? And can anyone come and help, please? [Or] is anyone off that could come in for a couple of hours, please? Just desperate, you know, double overtime, whatever overtime or pay, whatever, just please come and help us. Desperation is palpable. That's what they're doing. And the offers of people coming in has dwindled to nothing because nobody can do it anymore. (S7_MW_07) |

Key: MW = Midwife DR = Doctor HOS = Head of Service SU = Service User STMW = Student Midwife
