# Supplementary material for: ‘There's only so much you can be pushed’: Magnification of the maternity staffing crisis by the 2020/21 COVID‐19 pandemic
Source: BJOG. 2022 May 26;129(8):1408–9. doi: 10.1111/1471-0528.17203 (PMC9321880; doi:10.1111/1471-0528.17203)
Supplement: Supplementary file 2 — File S2 [file BJO-129-1408-s006.docx]

### Members of the ASPIRE COVID-19 Collaborative Group

#### Co-investigators

Soo Downe, University of Central Lancashire; George Ellison, University of Central Lancashire; Alan Fenton, Newcastle upon Tyne Hospitals NHS Foundation Trust; Alexander Heazell, University of Manchester; Ank de Jonge, Amsterdam University Medical Center; Carol Kingdon, University of Central Lancashire; Sarah Neal, University of Southampton; Zoe Matthews, University of Southampton; Alexandra Severns, NHS England and NHS Improvement North West; Gill Thomson, University of Central Lancashire; Anastasia Topalidou, University of Central Lancashire; Alison Wright, Royal Free Teaching Hospital in London.

#### Research Staff

Naseerah Akooji, University of Central Lancashire; Marie-Clare Balaam, University of Central Lancashire; Jo Cull, University of Central Lancashire; Lauri van den Berg, Amsterdam University Medical Center; Sarah Cordey, University of Central Lancashire; Nicola Crossland, University of Central Lancashire; Claire Feeley, University of Central Lancashire; Beata Franso, Amsterdam University Medical Center; Steph Heys, University of Central Lancashire; Zoe Matthews, University of Southampton; Gill Moncrieff, University of Central Lancashire; Sarah Neal, University of Southampton; Rebecca Nowland, University of Central Lancashire; Deborah Powney, University of Central Lancashire; Arni Sarian, University of Central Lancashire; Lucy Stone, University of Southampton; Heidi Tranter, University of Central Lancashire; Joanne Harris, University of Central Lancashire.

#### Steering committee

Maria Booker, Birthrights; Jane Sandall, Kings College London; Jim Thornton (chair), The University of Nottingham; Tisian Lynskey-Wilkie, University of Central Lancashire; Vanessa Wilson, East Lancashire Hospitals NHS Trust.

**Advisory Group**

Rebecca Abe and Tinuke Awe, FivexMore; Toyin Adeyinka, MVP BAME Group; Ruth Bender-Atik, The Miscarriage Association; Lia Brigante, RCM; Rebecca Brione, Birthrights; Franka Cadée, International Confederation of Midwives (ICM); Elizabeth Duff, Expert; postnatal care; Tim Draycott, Royal College of Obstetricians and Gynaecologists (RCOG); Duncan Fisher, Fathers included/Family included/the Family Initiative; Annie Francis, Neighbourhood Midwives; Arie Franx, Erasmus MC; Lucy Frith, University of Liverpool; Louise Griew, National Maternity Voices; Clea Harmer, SANDS; Caroline Homer, Burnet Institute; Australia; Marian Knight, National Perinatal Epidemiology Unit (NPEU); Amali Lokugamage, University College London; Amanda Mansfield, London Ambulance Service Trust; Neil Marlow, University College London; Trixie Mcaree, NHS England; David Monteith, Grace in Action; Keith Reed, Twins Trust; Yana Richens, UCL & City University; Lucia Rocca-Ihenacho, Midwifery Unit Network; Mary Ross-Davie, RCM Scotland; Seana Talbot, BirthWise NI; Myles Taylor, British Maternal and Fetal Medicine Society; Maureen Treadwell, Birth Trauma Association.

**Local PIs**

Katrina Rigby, Gillian Houghton, Claire Worthington, Clare O’Brien, James Harris, Ajith Wijesiriwardana, Heidi Hollands

**Recruiting Centres**

Lancashire Teaching Hospitals NHS Foundation Trust (Katrina Rigby, Cheryl Wyatt, Julie Earnshaw); Liverpool Women’s NHS Foundation Trust (Gillian Houghton, Amy Mahdi, Caroline Cunningham, Michelle Dower, Siobhan Holt, Sian Rodgers); Royal Surrey NHS Foundation Trust (Claire Worthington, Caroline Eynon, Michelle Maunder, Paula Lavandeira Fernandez); South Warwickshire NHS Foundation Trust (Clare O'Brien, Sandra Murphy, Kelly Jukes): Chelsea and Westminster NHS Trust (James Harris, Georgia Contosorou):University Hospitals Plymouth NHS Trust (Heidi Holland, Donna Underwood); North Cumbria NHS Trust (Ajith Wijesiriwardana, Theresa Cooper, Rachel Hardy, Anna McSkeane, Adrian Hodgson)
